# The moderating effect of mental health and health insurance ownership on the relationships between physical multimorbidity and healthcare utilisation and catastrophic health expenditure in India

**DOI:** 10.1186/s12877-023-04531-8

**Published:** 2024-01-03

**Authors:** Finja Berger, Kanya Anindya, Sanghamitra Pati, Shishirendu Ghosal, Stefanie Dreger, John Tayu Lee, Nawi Ng

**Affiliations:** 1 School of Public Health and Community Medicine, Institute of Medicine, Sahlgrenska Academy, University of Gothenburg, Gothenburg, Sweden; 2 ICMR-Regional Medical Research Centre, Bhubaneswar, Odisha India; 3 Institute of Public Health and Nursing Research, Department of Social Epidemiology, University of Bremen, Bremen, Germany; 4 College of Health and Medicine, Australian National University, Canberra, ACT Australia

**Keywords:** Mental health, Physical multimorbidity, Health insurance, Moderation, LASI, India, Healthcare utilisation, Catastrophic health expenditure

## Abstract

**Background:**

The current demographic transition has resulted in the growth of the older population in India, a population group which has a higher chance of being affected by multimorbidity and its subsequent healthcare and economic consequences. However, little attention has been paid to the dual effect of mental health conditions and physical multimorbidity in India. The present study, therefore, aimed to analyse the moderating effects of mental health and health insurance ownership in the association between physical multimorbidity and healthcare utilisation and catastrophic health expenditure (CHE).

**Methods:**

We analysed the Longitudinal Aging Study in India, wave 1 (2017–2018). We determined physical multimorbidity by assessing the number of physical conditions. We built multivariable logistic regression models to determine the moderating effect of mental health and health insurance ownership in the association between the number of physical conditions and healthcare utilisation and CHE. Wald tests were used to evaluate if the estimated effects differ across groups defined by the moderating variables.

**Results:**

Overall, around one-quarter of adults aged 45 and above had physical multimorbidity, one-third had a mental health condition and 20.5% owned health insurance. Irrespective of having a mental condition and health insurance, physical multimorbidity was associated with increased utilisation of healthcare and CHE. Having an additional mental condition strengthened the adverse effect of physical multimorbidity on increased inpatient service use and experience of CHE. Having health insurance, on the other hand, attenuated the effect of experiencing CHE, indicating a protective effect.

**Conclusions:**

The coexistence of mental health conditions in people with physical multimorbidity increases the demands of healthcare service utilisation and can lead to CHE. The findings point to the need for multidisciplinary interventions for individuals with physical multimorbidity, ensuring their mental health needs are also addressed. Our results urge enhancing health insurance schemes for individuals with mental and physical multimorbidity.

**Supplementary Information:**

The online version contains supplementary material available at 10.1186/s12877-023-04531-8.

## Introduction

Multimorbidity, characterised by the occurrence of two or more long-term conditions within an individual [1], is an escalating global health concern [2]. India, the world’s most populous country, is confronted with a major demographic transition: the population aged 60 and above is expected to more than double from slightly above 150 million in 2023 to around 347 million in 2050 [3]. The burden of multimorbidity grows along with ageing populations [4], as in India, encompassing a wide range of health conditions that can be related or unrelated, including both physical and mental health conditions [5]. This complex health profile places individuals with multimorbidity at higher risk of encountering challenges in effectively managing their health [5].

Patients with multimorbidity, especially those with both mental and physical conditions, have complex health needs [5], which require the significant use of healthcare services [6, 7]. Moreover, since multimorbidity is a lifelong condition, these individuals face higher healthcare expenditures and a higher likelihood of catastrophic health expenditure (CHE) [6, 8, 9]. Multimorbidity also poses a significant impact on work productivity, pushing affected patients and their families into poverty [10].

Health insurance is a potential mechanism for protecting people from experiencing CHE by enabling individuals to receive healthcare [11, 12] and reducing their financial burdens [12]. In India, various public, private and community-based insurance schemes coexist [13, 14]. Several governmental protection schemes have been introduced, including *Rashtriya Swasthya Bima Yojana* (RSBY), rolled out in 2008 for households below the poverty line [15]. To accelerate progress towards Universal Health Coverage (UHC) and Sustainable Development Goal (SDG) Target 3.8 – which ensures quality health services according to people’s needs while preventing financial hardship [16], the *Ayushman Bharat* scheme was introduced in 2018. The scheme is comprised of two components: 1) Health and Wellness Centers to deliver preventive and promotive care and 2) *Pradhan Mantri Jan Arogya Yojana* (PM-JAY), the world’s largest government-funded insurance scheme, which is intended to cover secondary and tertiary care for 550 million beneficiaries [17]. However, in 2019–21, only 41% of households had at least one member covered by health insurance [18] and 50.6% of the total health expenditure was borne out-of-pocket (OOP) in 2020 due to insufficient public spending on health [19].

While several studies have explored the economic implications of multimorbidity, only a few have assessed the combined effect of physical and mental health conditions in individuals with multimorbidity. Findings indicate that additional mental health conditions in individuals with physical multimorbidity exacerbate adverse outcomes on healthcare utilisation [10, 20] and are strongly associated with CHE [21]. However, there is a critical knowledge gap in India concerning the potential role of health insurance in the relationship between physical and mental health conditions, healthcare utilisation, and CHE. Thus, this study aims to bridge this evidence gap by assessing the moderating effects of physical multimorbidity, mental health and health insurance ownership with healthcare utilisation and CHE in India.

## Methods

### Data and study population

This study utilised data from the first wave of the Longitudinal Aging Study in India (LASI), version B. LASI was conducted during 2017–2018 in all Indian states and union territories, except Sikkim, where data collection was carried out between 2020 and 2021. LASI aims to provide a baseline for ageing research to inform policy and advance scientific knowledge by supplying information on the disease burden, functional health, healthcare, and the social and economic well-being of older people in India. A multistage stratified area probability cluster sampling was implemented to ensure a nationally representative sample selection, considering three and four stages in rural and urban areas, respectively. The overall targeted sample size was 61,000 households. In version B of the LASI data, information of 73,396 adults aged 45 or above and their spouses, regardless of age, residing in the same household in all states and union territories across India are provided. Detailed information about the sampling process can be reviewed elsewhere [13].

Data from the household, the individual, and the biomarker survey were extracted and linked together for the analysis. Out of the total 73,396 respondents, 63,161 were included in the study. We excluded a duplicate record (*n* = 1), individual records with missing data on the household survey (*n* = 1126), respondents aged below 45 years (*n* = 6701) and proxy interviews (*n* = 696). After this, 1711 records were further excluded due to missing data on one or more variables included in the analysis. Detailed information on missing data is provided in the Additional file 1: Table S2 and S3. We decided to exclude people aged below 45 years since non-communicable diseases (NCDs) usually occur among people 45 years onwards [22]. We also excluded proxy interviews since they tend to yield inaccurately reported usage of healthcare services [23], probable invalid reporting of stigmatised health conditions, especially mental illnesses, and potentially inaccurate reporting of symptoms related to depression.

### Study variables

#### Independent variables

Physical and mental conditions were considered in assessing the respondent’s health status. For physical conditions, we focused on NCDs. We included the following 11: hypertension, diabetes, cancer/malignant tumours, chronic lung diseases, chronic heart diseases, stroke, musculoskeletal disorders, neurological/degenerative diseases (i.e., Alzheimer’s or Dementia, Parkinson), high cholesterol, thyroid disorder and chronic kidney diseases (chronic renal failure, kidney stones, benign prostate hyperplasia). The respondents were asked if they had ever been diagnosed with these chronic conditions. Along with self-reported hypertension, we considered the mean of the last two systolic and diastolic blood pressure readings (≥140 mmHg systolic blood pressure and/or ≥ 90 mmHg diastolic blood pressure) [24]. Due to data confidentiality considerations, we counted the number of physical conditions and differentiated between respondents having 0, 1, 2, or 3+ physical conditions. People with at least two physical conditions were considered as having physical multimorbidity.

We used self-reported diagnoses of depression or other psychiatric disorders, such as unipolar/bipolar disorder or schizophrenia, to identify respondents with a mental health condition. Further, we also used two composite scales based on self-reported depression symptoms, the CES-D-10 and CIDI-SF, to measure depressive disorder. The LASI survey included the short – 10-question – version of the Center for Epidemiologic Studies Depression Scale (CES-D-10 Scale) [25] to capture people with depressive symptoms. The CES-D-10 Scale caught whether the respondents experienced seven negative and three positive symptoms during the past week. For negative symptom questions, response options “rarely or never”/ “sometimes” were scored zero, while “often”/“most or all of the time” were scored one. Scoring was reversed for the positive symptom questions [13]. The overall depressive symptom score was calculated as the sum of the scores, and a cut-off of four or more was used to assess probable depressive symptoms [26].

Further, the Short Form Composite International Diagnostic Interview (CIDI-SF) Major Depression Episode [27] was used to capture major depressive disorder. Respondents endorsing the three screening questions for dysphoria received a set of seven symptom questions. Positive answers were scored as 1. The same structure was followed for anhedonia symptoms, except eligible respondents only received six symptom questions. The positive response to all screening questions was calculated as one additional point. A cut-off score of three or more for each dysphoria and anhedonia question set (range: 0–7) was used to identify respondents with probable major depressive symptoms [27]. In the LASI survey, no further distinction of the type of mental health condition, apart from depression and other psychiatric disorders, was considered. Overall, respondents were categorised as suffering from a mental health condition when they either self-reported the disorders mentioned above and/or were classified as depressed by one of the composite scales.

Respondents were asked whether they were covered by health insurance through reimbursement or direct payment for medical or surgical expenses. Based on this, we constituted a binary variable for assessing the status of health insurance ownership (no health insurance/owned health insurance).

#### Outcome variables

Healthcare utilisation was assessed based on self-reported questions for inpatient and outpatient care. For inpatient care, respondents were asked about the number of admissions for at least one night to a hospital/long-term care facility in the past 12 months when they indicated that they had visited one in the past 12 months. Respondents who have not visited any hospital/long-term care facility or have not been admitted for at least one night were considered as not having received inpatient care. The assessment of outpatient care followed a similar logic. It was based on the questions of whether the respondent has consulted any healthcare provider in the past 12 months and, for those who have, the number of times they received healthcare or consultation (including home visits). Inpatient and outpatient care were coded as dummy variables (0 = No; 1 = Yes).

To assess CHE, the exceedance of the total health expenditure of the household of every respondent (THEALTH_H_) of certain thresholds was measured. Three different recommendations were used to assess the robustness of the results: 10% and 25% thresholds in relation to the total household expenditure (THE_H_) as proposed by the SDGs [28] and a 40% threshold related to the household’s capacity to pay (CTP_H_) [29]. THE_H_ reflects the sum of the food, healthcare and other non-food expenditures on the household level. CTP_H_ is the total household expenditure excluding basic subsistence needs [30] and is proxied by THE_H_, excluding food expenditures [31]. All expenditure variables were aggregated to annual consumption. According to these definitions, people were categorised as experiencing CHE when $\frac{{THE AlTH}_H}{THE_H}>z$ or $\frac{THEALTH_H}{CTP_H}>z$ whereby z indicates the thresholds. For the financial variables, we used imputations based on the University of Southern California method, which considers other information provided by the household or information supplied by comparable households to address missing values [32].

#### Covariates

Sociodemographic and socioeconomic factors included were: sex (male/female), age (45–59 years, 60–74 years, 75 and above years), residence (rural/urban), marital status (not married or in a relationship/married or in a relationship), religion (Hindu/Muslim/Christian/Sikh/Other or none), social group (scheduled tribe, scheduled caste, other backward class, other/no social group), highest attained educational level (no education/up to primary/middle school to higher secondary/diploma, graduation and above), employment status (never worked/currently not working but worked before/currently working), and total household expenditure per capita categorised in quintiles (Q1 (the lowest) - Q5 (the highest)). The selection was informed by previous studies assessing the associations between covariates, multimorbidity and healthcare utilisation/expenditure (e.g. [33–37]).

### Statistical analyses

The proportions of respondents using outpatient/inpatient care and experiencing CHE were assessed for populations within different socioeconomic and sociodemographic groups. We also presented the proportion of healthcare utilisation and experience of CHE for the population stratified by their physical conditions, ownership of health insurance and mental health condition. We estimated the moderation effects between the number of physical conditions, mental health condition and ownership of health insurance on healthcare utilisation and CHE using multivariable logistic regression, adjusted for: age, sex, place of residence, marital status, religion, social group, education, employment status, household expenditure per capita. The model can be expressed as follows:

$$\text{logit}\left(p\right)=\mathit{\log}\left(\frac{p}{1-p}\right)={\beta}_0+{\beta}_1 physical+{\beta}_2 mental+{\beta}_3 insurance+{\beta}_4\left( physical\times mental\right)+{\beta}_5\left( physical\times insurance\right)+{\beta}_6\left( mental\times insurance\right)+{\beta}_7\left( physical\times mental\times insurance\right)+\left({\sum}_{n=1}^C{\beta}_{n+7}{X}_n\ \right)$$

where *p* denotes the probability of having the outcome, *β*_0_ represents intercept, *X*_*n*_ represents covariates with C being the number of covariates. We only reported the moderation effects in the table to avoid misinterpretation of the estimates of the covariates, known as the “Table 2 fallacy” [38]. Comprehensive tables presenting unadjusted odds ratio are provided in the Additional file 1: Tables S4 and S5. Wald tests were conducted to assess the significant difference in the estimates between different combinations of stratification variables. Finally, we calculated the predicted probability for healthcare utilisation and CHE for each of the combinations of the stratification variables using the margins command in Stata SE 17.0. We plotted the estimated probability using the *marginsplot* command. All analyses were weighted with LASI’s sample weights, considering and adjusting selection probabilities, non-responses and post-stratification [32].

## Results

Table 1 presents the sample distribution and weighted prevalence of physical and mental morbidities. There were more females than males in the study population (53.6% vs. 46.4%). Around half of the respondents were between 45 and 59 years, 50.6% did not have education, almost 70% resided in rural areas, and 79.5% did not own health insurance. Around 38.6% of the respondents had no physical condition, while nearly one-quarter had physical multimorbidity. Almost one-third of the respondents reported and/or were identified through one of the composite scales as having a mental health condition.

**Table 1:** Baseline characteristics and prevalence (%) of healthcare utilisation and experience of catastrophic health expenditure

	Total		Prevalence of healthcare utilisation (%)		Prevalence of catastrophic health expenditure (%)		
	Freq.	%	Outpatient	Inpatient	> 10 of THE_H_	> 25 of THE_H_	> 40 of CTP_H_
**Total**	63,161		57.3	7.0	33.1	12.3	18.3
**Sex**							
Male	29,281	46.4	54.4	7.3	33.0	11.9	17.6
Female	33,880	53.6	59.8	6.7	33.2	12.6	18.8
**Age**							
45–59 years	31,667	50.1	54.4	6.0	30.3	10.3	14.8
60–74 years	24,618	39.0	60.5	7.6	34.9	13.8	21.1
75+ years	6876	10.9	59.2	8.7	39.5	15.7	24.0
**Residence**							
Rural	44,115	69.8	57.9	6.7	34.3	13.0	20.2
Urban	19,046	30.2	55.9	7.5	30.2	10.6	13.8
**Marital status**							
Not married or in a relationship	16,068	25.4	59.6	7.3	30.5	11.4	18.1
Married or in a relationship	47,093	74.6	56.5	6.9	34.0	12.5	18.4
**Religion**							
Hindu	52,101	82.5	56.4	6.6	32.3	12.2	17.9
Muslim	7065	11.2	64.3	9.0	40.2	12.7	21.3
Christian	1741	2.8	47.5	7.3	24.6	9.3	13.7
Sikh	1150	1.8	72.3	7.1	39.7	14.5	20.5
Other or none	1104	1.7	53.5	8.4	32.2	13.6	20.0
**Social group**							
Scheduled tribe	5480	8.7	44.4	5.7	20.5	7.3	13.1
Scheduled caste	12,395	19.6	59.2	7.4	35.0	12.9	20.8
Other backward class	28,568	45.2	56.7	7.0	32.7	12.1	17.5
Other or no caste/tribe	16,719	26.5	61.2	7.0	36.5	13.7	19.4
**Educational level**							
No education	31,961	50.6	57.8	7.0	33.3	12.2	20.2
Up to primary	14,725	23.3	59.4	8.2	33.8	13.5	18.2
Middle school to higher secondary	12,852	20.3	55.3	6.4	32.9	12.0	15.8
Diploma, graduation or above	3623	5.7	51.4	3.9	29.1	8.8	10.6
**Employment status**							
Never worked	16,020	25.4	56.0	6.2	32.9	12.8	18.8
Currently not working but worked before	17,196	27.2	62.9	11.1	38.0	15.3	22.1
Currently working	29,946	47.4	54.8	5.0	30.4	10.2	15.8
**Household expenditure per capita**							
Q1 (the lowest)	13,819	21.9	51.6	4.6	23.4	6.4	14.6
Q2	13,442	21.3	56.3	5.2	30.2	8.7	16.8
Q3	12,584	19.9	59.6	7.0	36.4	12.5	20.6
Q4	11,731	18.6	61.0	8.2	36.9	15.2	19.1
Q5 (the highest)	11,585	18.3	59.1	10.6	40.6	20.1	21.1
**Number of physical conditions**							
0	24,353	38.6	49.3	3.9	28.0	9.6	15.7
1	23,072	36.5	57.2	6.4	31.5	11.9	17.7
2	10,938	17.3	68.8	10.9	42.0	16.0	21.7
3+	4799	7.6	72.4	16.3	46.4	19.1	26.5
**Mental health condition**							
No mental health condition	43,241	68.5	56.5	5.7	30.5	10.5	15.9
With mental health condition	19,920	31.5	59.0	9.7	38.7	16.0	23.4
**Ownership of health insurance**							
No health insurance	50,193	79.5	57.1	6.6	33.9	12.6	19.0
Owned health insurance	12,968	20.5	58.1	8.2	30.1	10.8	15.6

Estimates are weighted. | *THE*_*H*_ total household expenditure, *CTP*_*H*_ capacity to pay

### Healthcare utilisation

Around 57% and 7% of the respondents used outpatient and inpatient services at least once in the past 12 months, respectively (Table 1). The prevalence of inpatient and outpatient care utilisation increased with more physical conditions (Table 1), irrespective of having a mental condition and/or health insurance (Table 2). Further, among respondents with the same physical and mental health burden, those with health insurance generally showed higher utilisation rates. Likewise, inpatient services were more prevalent among those with a mental health condition than respondents without one, irrespective of their physical conditions or ownership of health insurance (Table 2).

**Table 2:** Prevalence (%) of healthcare utilisation and experience of catastrophic health expenditure for stratification variables

Number of physical conditions	No health insurance		Owned health insurance	
	No mental health condition	With mental health condition	No mental health condition	With mental health condition
**Prevalence of outpatient care utilisation (%)**				
0	49.2	51.4	46.0	51.2
1	56.3	57.7	58.0	60.1
2	68.5	67.9	69.8	72.9
3+	73.4	68.8	78.4	71.0
**Prevalence of inpatient care utilisation (%)**				
0	3.2	4.6	4.3	7.2
1	5.6	7.9	5.7	8.5
2	8.1	15.4	9.5	15.4
3+	11.7	17.9	18.5	31.0
**Prevalence of catastrophic health expenditure - CHE > 10 of THE**_**H**_ **(%)**				
0	26.9	32.9	25.2	26.2
1	30.1	37.9	25.5	31.2
2	39.7	49.0	36.7	40.8
3+	42.1	52.5	43.2	52.3
**Prevalence of catastrophic health expenditure - CHE > 25 of THE**_**H**_ **(%)**				
0	8.6	12.5	9.0	9.0
1	10.6	16.4	8.4	12.6
2	14.2	21.0	11.8	18.2
3+	16.4	23.7	17.8	18.9
**Prevalence of catastrophic health expenditure - CHE > 40 of CTP**_**H**_ **(%)**				
0	14.7	19.9	13.3	13.6
1	16.3	23.3	12.8	18.9
2	18.6	29.4	15.6	25.0
3+	22.7	33.3	22.2	28.7

Estimates are weighted. | *CHE* catastrophic health expenditure, *THE*_*H*_ total household expenditure, *CTP*_*H*_ capacity to pay

For individuals with no mental health condition and no health insurance, the adjusted odds of outpatient and inpatient care utilisation increased by 1.41 [95% CI = 1.33–1.49] and 1.37 [95% CI = 1.28–1.47] times with one unit increase in the number of physical conditions, respectively. For those with a mental condition and no health insurance, the adjusted odds ratio was 1.35 [95% CI = 1.28–1.42] for outpatient care and 1.74 [95%CI = 1.60–1.89] for inpatient care. The lower adjusted odds ratio for outpatient than that observed in inpatient care was similar for those with a mental condition and owned health insurance, with the corresponding odds ratio of 1.46 [95% CI = 1.35–1.59] and 2.02 [95% CI = 1.76–2.31], respectively (Table 3). A pairwise comparison of the regression coefficients showed a statistically significant difference between individuals with a mental condition and no health insurance and those without a mental condition who owned health insurance (Wald Test *p*-value< 0.01) for outpatient care. Significant differences in inpatient care utilisation were evident for multiple comparisons between respondents without and with mental condition when they either owned or did not own health insurance (Additional file 1: Table S6).

**Table 3:** Logistic regression models for healthcare utilisation

	Healthcare utilisation			
	Outpatient		Inpatient	
	OR [95% CI]	AOR^a^ [95% CI]	OR [95% CI]	AOR^a^ [95% CI]
Physical conditions # No mental health condition # No health insurance	1.43 [1.35–1.52]	1.41 [1.33–1.49]	1.46 [1.37–1.56]	1.37 [1.28–1.47]
Physical conditions # No mental health condition # Owned health insurance	1.52 [1.40–1.66]	1.54 [1.42–1.66]	1.66 [1.52–1.81]	1.56 [1.43–1.70]
Physical conditions # With mental health condition # No health insurance	1.40 [1.33–1.48]	1.35 [1.28–1.42]	1.90 [1.70–2.13]	1.74 [1.60–1.89]
Physical conditions # With mental health condition # Owned health insurance	1.50 [1.38–1.63]	1.46 [1.35–1.59]	2.18 [1.89–2.52]	2.02 [1.76–2.31]

Estimates are weighted | *OR* odds ratio, *AOR* adjusted odds ratio

^a^Adjusted for: age, sex, place of residence, marital status, religion, social group, education, employment status, household expenditure per capita

**Fig. 1:**
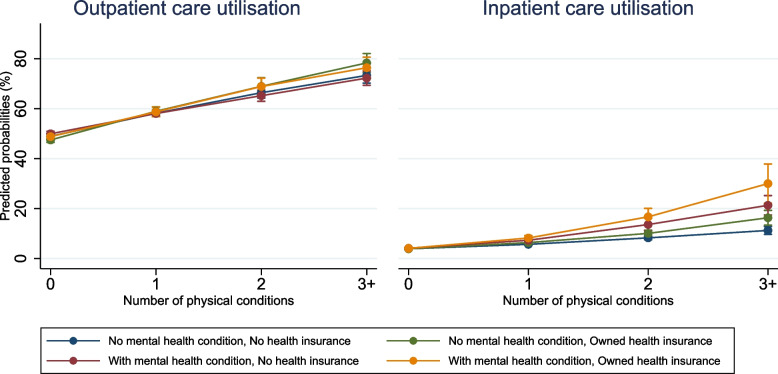
Adjusted predicted probabilities for healthcare utilisation with 95% CIs Adjusted for: sex, age, place of residence, marital status, religion, education, employment status, household expenditure per capita. Estimates are weighted

Among people without any physical condition, the predicted probability for outpatient care utilisation ranged from 47.5% [95% CI = 46.5–48.5] among those without a mental health condition and owning health insurance to 49.9% [95% CI = 49.0–50.9] among respondents with a mental health condition and no health insurance. However, with the increasing number of physical conditions, the predicted probability for outpatient care utilisation increased significantly for individuals with 3+ conditions. It ranged from 72.2% [95% CI = 69.3–75.2] among those with a mental health condition and not owning health insurance to 78.3% [95% CI = 74.5–82.1] among respondents without a mental health condition but owning health insurance. For inpatient care, the predicted probability was nearly similar across the stratification groups for zero conditions. With increasing physical conditions, however, the predicted probability was constantly lowest for respondents with neither a mental health condition nor health insurance and highest for respondents with a mental health condition and health insurance. The difference between these groups was almost 20 percentage points for 3+ conditions (11.2% vs 30%) (Fig. 1; Additional file 1: Table S7).

### Catastrophic health expenditure

Based on the 10% and 25% THE_H_ and the 40% CTP_H_ definitions, 33.1%, 12.3% and 18.3% of the respondents were categorised as experienced CHE, respectively (Table 1). Irrespective of the definition used, a general trend in the proportion of respondents who experienced CHE was observed in the stratified analyses, with just some slight inconsistencies (Table 2). The proportion of people experiencing CHE increased with the number of physical conditions irrespective of health insurance ownership and mental health condition (Table 2). When comparing individuals with the same number of physical conditions and health insurance ownership, more respondents with a mental health condition reported experiencing CHE than those without a mental health condition. A similar pattern was evident when comparing those with or without a mental health condition among those who owned and did not own health insurance.

The adjusted odds ratio of experiencing CHE was lowest for respondents without mental health condition but owned health insurance, irrespective of the CHE’s definition used (Table 4). In contrast, the respondents with mental health condition but no health insurance showed the highest odds of experiencing CHE. For each increase in the number of physical conditions, they were 1.40 times [95%CI = 1.33–1.48] more likely to experience CHE for 40% CTP_H_ and 1.46 times [95%CI = 1.38–1.53] for 10% THE_H_. Apart from that, most pairwise comparisons of the moderating effects of mental health condition and ownership of health insurance on physical conditions differed significantly (Additional file 1: Table S6).

**Table 4:** Logistic regression models for the experience of catastrophic health expenditure

	Catastrophic health expenditure					
	> 10% of THE_H_		> 25% of THE_H_		> 40% of CTP_H_	
	OR [95% CI]	AOR^a^ [95% CI]	OR [95% CI]	AOR^a^ [95% CI]	OR [95% CI]	AOR^a^ [95% CI]
Physical conditions # No mental health condition # No health insurance	1.25 [1.18–1.33]	1.20 [1.14–1.27]	1.21 [1.14–1.29]	1.11 [1.05–1.18]	1.13 [1.07–1.19]	1.10 [1.05–1.16]
Physical conditions # No mental health condition # Owned health insurance	1.18 [1.06–1.31]	1.14 [1.04–1.25]	1.13 [1.04–1.23]	1.04 [0.96–1.13]	1.02 [0.95–1.10]	1.02 [0.95–1.10]
Physical conditions # With mental health condition # No health insurance	1.53 [1.45–1.62]	1.46 [1.38–1.53]	1.54 [1.46–1.63]	1.41 [1.33–1.49]	1.48 [1.40–1.56]	1.40 [1.33–1.48]
Physical conditions # With mental health condition # Owned health insurance	1.37 [1.25–1.49]	1.34 [1.23–1.45]	1.35 [1.23–1.49]	1.27 [1.15–1.39]	1.31 [1.20–1.44]	1.29 [1.18–1.41]

Estimates are weighted. | *THE*_*H*_ total household expenditure, *CTP*_*H*_ capacity to pay, *OR* odds ratio, *AOR* adjusted odds ratio

^a^Adjusted for: age, sex, place of residence, marital status, religion, social group, education, employment status, household expenditure per capita

**Fig. 2:**
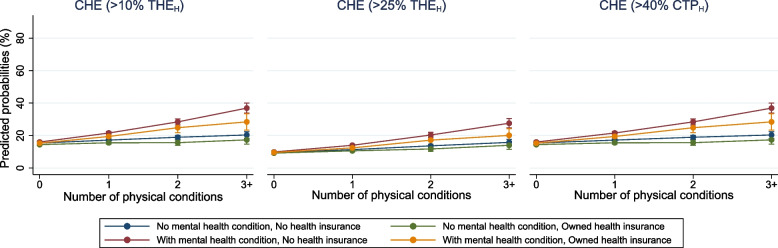
Adjusted predicted probabilities for the experience of catastrophic health expenditure with 95% CIs Adjusted for: sex, age, place of residence, marital status, religion, education, employment status, household expenditure per capita. Estimates are weighted. | CHE = catastrophic health expenditure; THEH = total household expenditure; CTPH = capacity to pay

Regardless of the number of physical conditions, the predicted probability of experiencing CHE was consistently highest for those with an additional mental health condition but no health insurance and lowest for those without mental health condition and who had health insurance. The predicted probability of experiencing CHE increased with the increasing number of physical conditions. These patterns were observed consistently across the different CHE thresholds (Fig. 2).

For example, among respondents with zero physical conditions, the predicted probabilities of experiencing CHE at 10% THE_H_ threshold were 26.2% [95%CI = 25.4–27.0] for those without a mental health condition and owned health insurance and 27.8% [95%CI = 26.9–28.6] for those with mental health condition but no health insurance. While among individuals with 3+ physical conditions, the predicted probabilities increased to 39.1% [95%CI = 32.4–45.7] for those without mental health condition and owned health insurance and to 57.2% [95%CI = 53.9–60.6] for those with mental health condition but without health insurance. The difference in percentage points between the aforementioned highest and lowest groups with 3+ physical conditions ranged between 13.6% and 19.6%, depending on the CHE threshold (Additional file 1: Table S7).

## Discussion

The study aimed to assess the moderating effect of mental health and health insurance ownership in the association between physical multimorbidity and the utilisation of healthcare and experience of CHE. This study reveals (1) that burdens of healthcare utilisation and CHE are heterogeneous among individuals with physical multimorbidity, depending on their ownership of health insurance and their mental health, (2) that having an additional mental health condition strengthens the adverse effect, and (3) that health insurance might have the potential to ease adverse effects.

The study confirms the evidence from low and middle-income countries and India regarding the increased use of healthcare among people with physical multimorbidity [6, 7, 39]. The difference in the estimated effects for individuals with physical multimorbidity and varying mental health condition and health insurance ownership was marginal for outpatient care, whereas it was more substantial for inpatient care. Having an additional mental health condition to physical multimorbidity accelerated the use of inpatient services. Our finding might be explained by poor mental health care infrastructure [14] and the stigma surrounding mental illnesses in India. A qualitative study using focus group discussion in India illustrated that people with mental health conditions are often seen as weak, untidy, harmful, and a nuisance to the public. These perceptions result in less support for people with mental illness, leading them to hide their disease [40]. Besides, comorbidities of mental and physical conditions often complicate treatment-seeking and adherence, leading to delayed diagnosis and a worsened prognosis for the physical condition [41]. Further, although NCD and mental health programmes were implemented in India [42], the integration of multisectoral and integrated perspectives is limited [43]. Mental and physical health service programmes have traditionally been designed as vertical programmes [44]. Therefore, the additional health system barriers might limit people with mental health conditions from seeking help from outpatient services in the first place, worsening their condition. Consequently, they might seek treatment when their health state is severe and thus must receive inpatient care. Therefore, it is also imperative to strengthen the primary healthcare system by sensitising and improving the health system workforce and the community’s awareness of mental health issues [45].

Our findings reveal another intriguing aspect: the relationship between physical multimorbidity, mental health condition, and health service utilisation is more pronounced among individuals with health insurance compared to those without. This finding suggests that health insurance plays a crucial role in reducing the financial barriers to accessing necessary healthcare, resulting in lower unmet healthcare needs and a higher rate of doctor visits for patients with health conditions [46].

The findings also highlight that respondents with physical multimorbidity had a higher likelihood to experience CHE unconditionally on their mental health or ownership of health insurance, consistent with prior literature reporting higher health expenses and CHE in individuals with multimorbidity [6, 8, 9]. Having an additional mental health condition further strengthened the adverse outcome. When comparing people with physical multimorbidity with and without an additional mental health condition, individuals with a mental health condition had a stronger association for experiencing CHE. This might likely be explained by the reliance on inpatient care, which is normally more costly than outpatient services [47]. This could also be due to the reliance on private health services due to stigma and fear of accessing public services, and, hence, more need for privacy and confidentiality [48].

The findings further reveal the potential of health insurance, as the relationship between physical multimorbidity, mental health condition, and CHE is attenuated among individuals with health insurance despite their higher level of health service utilisation. Nevertheless, ownership of health insurance could not fully protect beneficiaries from financial burdens as our findings still illustrate a strong association for CHE among people with physical multimorbidity. This might indicate inadequate functioning of health insurance in India, working inefficiently in reducing the burden of catastrophic expenses [49]. It should be noted that the PM-JAY scheme was launched in 2018, implying that more vulnerable older people aged 45+ were not covered by any health insurance scheme when the first wave of LASI was conducted (2017–18). However, governmental schemes encounter several impediments, including the ineffectiveness of the RSBY scheme in protecting beneficiaries against OOP expenses [50], lack of awareness of insurance entitlement for PM-JAY [51], or delayed reimbursement forcing hospitals to ask patients to purchase medications from outside of the facility, resulting in OOP spending [52]. Therefore, the factors associated with the limited impact of health insurance schemes on health expenditures should be scrutinised thoroughly. Following enhancing the benefit packages and coverage for public and private healthcare providers, the national health policy should also address strategies to maintain equitable access to quality services, improve awareness, and strengthen enrolment.

### Strengths and limitations

This study analysed a nationally representative large-scale database that allows for the generation of evidence on healthcare utilisation and experience of CHE in India. Hereby, particular emphasis has been drawn on the moderating role of mental health and health insurance on healthcare utilisation and CHE among individuals with physical multimorbidity, which, to our knowledge, has not been done in the Indian context. This study contributes to understanding the role of health insurance ownership in buffering the effects of physical multimorbidity on CHE, particularly among individuals with physical multimorbidity and additional mental health conditions. This evidence supports the design of an intervention in clinical and primary healthcare settings to address mental health problems, especially depression or depressive symptoms, among individuals with physical multimorbidity. This study also contributes to increasing the understanding of healthcare utilisation and CHE in India, two important indicators for UHC, as one of the priorities in the SDGs.

However, the cross-sectional nature of this study does not allow for causal inference. Further, neither the number of visits nor the type of service or health insurance (public or private) was assessed in this study as we decided to portray the general role of health insurance. The findings, therefore, should be interpreted with caution. Most of the morbidity conditions included in this study, except for hypertension and depressive symptoms, were self-reported based on diagnoses made by health professionals, which are prone to underreporting due to lack of awareness of the diseases, limited healthcare access, and subsequently, underdiagnosis of the condition for those with barriers to healthcare access [53]. Trying to counteract this, we included blood pressure readings, the only publicly available objective measures, in hypertension diagnosis. We also measured depressive symptoms using composite scales, which have the potential to capture individuals with less severe depression that necessitate mental health care by healthcare professionals. However, the compositive scales for mental health condition were only available for depression but no other mental health conditions. Hence, the burden of mental health conditions might be underestimated in this study. Nevertheless, by considering depression, we have captured one of the most common mental disorders in India [54].

## Conclusion

The coexistence of mental health problem and physical multimorbidity poses a dual burden that necessitates more intense healthcare utilisation and a higher propensity to experience CHE. Our study reveals the buffering effect of health insurance on attenuating the effect of physical multimorbidity on healthcare utilisation and experience of CHE, particularly among individuals with the dual burden of physical multimorbidity and mental health. These results demonstrate the relevance of financial protection schemes to buffer the adverse outcomes associated with mental health conditions and physical multimorbidity but also illustrate the need to improve existing schemes as the economic burden remains high, even when owning health insurance. Nevertheless, only trying to reduce the economic burden via existing insurance systems might be insufficient as individuals with physical multimorbidity and a mental health condition showed stronger associations with experiencing CHE, regardless of having health insurance. Identifying barriers that limit patients with mental health conditions from receiving healthcare services, especially outpatient services, through a qualitative study is essential. The findings could help in designing a tailored, multidisciplinary intervention to improve access and healthcare utilisation, as well as to prevent the adverse economic impacts of the dual burden of physical multimorbidity and mental health problems.

Future research could consider multimorbidity as a heterogeneous condition and hence focus on different combinations of mental and physical conditions and compare their impacts on relevant healthcare utilisation and expenditure indicators. Future studies should also explore the role of public and private health services and specific types of insurance forms in mitigating the burdens of physical multimorbidity and mental health condition.

### Supplementary Information

**Additional file 1: Table S1. **Construction of variables from the LASI questionnaire. **Table S2.** Missing values per variable. **Table S3.** Number of variables with missing data per respondent. **Table S4.** Logistic regression models for healthcare utilisation. **Table S5.** Logistic regression models for the experience of catastrophic health expenditure. **Table S6.** Results of the Wald test between stratification groups for the estimates of the logistic regressions. **Table S7.** Predicted probabilities for healthcare utilisation and experience of catastrophic health expenditure.

## Data Availability

The present study utilised data from version B of The Longitudinal Aging Study in India (wave 1), 2017–2018. The study was produced by the International Institute for Population Sciences, Harvard T.H. Chan School of Public Health, and the University of Southern California and distributed by the University of Southern California with funding from the Ministry of Health and Family Welfare, Government of India, the National Institute on Aging (R01 AG042778, R01 AG030153), and United Nations Population Fund, India. The de-identified data of LASI wave 1 can be downloaded from the Gateway to Global Aging Data. [Gateway to Global Aging Data | LASI: Study Overview (g2aging.org)].

## Abbreviations

AOR: Adjusted odds ratio
CES-D: Center for Epidemiologic Studies Depression Scale
CHE: Catastrophic health expenditure
CIDI-SF: Short Form Composite International Diagnostic Interview
CTP_H_: Household’s capacity to pay
LASI: Longitudinal Aging Study in India
NCD: Non-communicable disease
OOP: Out-of-pocket
OR: Odds ratio
PM-JAY: Pradhan Mantri Jan Arogya Yojana
RSBY: Rashtriya Swasthya Bima Yojana
SDG: Sustainable Development Goal
THE_H_: Total household expenditure
THEALTH_H_: Total health expenditure on household level
UHC: Universal Health Coverage
