# Supplementary material for: The moderating effect of mental health and health insurance ownership on the relationships between physical multimorbidity and healthcare utilisation and catastrophic health expenditure in India
Source: BMC Geriatr. 2024 Jan 3;24:6. doi: 10.1186/s12877-023-04531-8 (PMC10762917; doi:10.1186/s12877-023-04531-8)
Supplement: Supplementary file 1 — Additional file 1: Table S1. Construction of variables from the LASI questionnaire. Table S2. Missing values per variable. Table S3. Number of variables with missing data per respondent. Table S4. Logistic regression models for healthcare utilisation. Table S5. Logistic regression models for the experience of catastrophic health expenditure. Table S6. Results of the Wald test between stratification groups for the estimates of the logistic regressions. Table S7. Predicted probabilities for healthcare utilisation and experience of catastrophic health expenditure. [file 12877_2023_4531_MOESM1_ESM.docx]

**Additional File**

| **Table S1:** Construction of variables from the LASI questionnaire. | | |
| --- | --- | --- |
| **Outcome variables** | | |
| ***Outpatient care utilisation*** | HC003: In the past 12 months, have you consulted any health care provider? [Please identify ALL healthcare providers that you have visited]   1. Doctor (with MBBS, including surgeon, physician, gynecologist, psychiatrist, ophthalmologist and orthopedician) 2. AYUSH practitioner (Ayurveda /unani/ siddha /homeopathy) 3. Dentist 4. Nurse/midwife 5. Physiotherapist 6. Pharmacist 7. Traditional /Folk healers (tribal medicine/bhopa/jhaad-fook/black magic) 8. Other, please specify 9. None   HC302: [Ask only if HC003<i] In the past 12 months, how many times did you receive healthcare or consultation from a healthcare  provider (including home visits)? | 1. No (HC003=i or any of HC003= a-h & HC302=0) 2. Yes (any of HC003= a-h & HC302 ≥ 1) |
| ***Inpatient care*** | HC002: In the past 12 months, have you visited any health care facility or any health professional has visited you? [Please identify ALL the facilities that you have visited]   1. Health post/sub centers 2. Primary health center/Urban Health Center 3. Community health center 4. District / Sub-district hospital 5. Government/tertiary hospital 6. Govt. AYUSH hospital 7. Private hospital/nursing home 8. Private clinic (OPD based services) 9. NGO/Charity/Trust/Church-run hospital 10. Private AYUSH hospital 11. . Health camp 12. Mobile healthcare unit 13. Pharmacy/drugstore 14. Home visit 15. Other, please specify 16. None   HC202: Over the last 12 months, how many times you were admitted as patient to a hospital/long-term care facility for at least one night? | 1. No (HC002=p or any of HC002=b-j & HC202=0) 2. Yes (any of HC002= b-j & HC202 ≥ 1) |
| *Outpatient care expenditure on household level* | *CO108—CO112. Next, think about your expenditure on outpatient health care in the past 30 days including expenses on medicines or tests, purchased with/without consulting a health care provider [do not include any expense which is reimbursed].* | *(Sum of CO108-CO112) * 12* |
| *Inpatient care expenditure on household level* | *CO202-CO207: In the past 12 months, how much did your household spend on the following health care expenses? Please report only inpatient visits (do not include any expense which is reimbursed).* | *Sum of CO202-CO207* |
| *Food expenditure on household level* | *CO002a-i: Please tell us about your household’s food consumption in the past 7 days, including not only market-purchased food, but also home-produced food and in-kind transfers* | *(Sum of CO002a-i) * 52* |
| *Regularly incurring non-food expenditure on household level* | *CO101 – CO107. In the past 30 days, approximately how much (in rupees) did your household spend on regularly recurring non-food expenditures?* | *Sum of CO101-CO107) * 12* |
| *Non-regularly incurring non-food expenditures* | *CO209 – CO216. In the past 12 months how much (in rupees) did your household spend on the following items?* | *Sum of CO209-CO216* |
| *Total health expenditure on household level (THEALTH_H_)* |  | *Sum of:*   - *Outpatient care expenditure* - *Inpatient care expenditure* |
| *Total household expenditure (THE_H_)* |  | *Sum of:*   - *Outpatient care expenditure* - *Inpatient care expenditure* - *Regularly occurring non-food expenditure* - *Irregularly occurring non-food expenditure* - *Food expenditure* |
| *Capacity to pay (CTP_H_)* |  | *Sum of:*   - *Outpatient care expenditure* - *Inpatient care expenditure* - *Regularly occurring non-food expenditure* - *Irregularly occurring non-food expenditure* |
| ***CHE 10 (>10% THE_H_)*** |  | 1. No CHE if $\frac{\mathrm{THEALTH}_{H}}{\mathrm{THE}_{H}}$ ≤ 0.1 2. CHE if $\frac{\mathrm{THEAlTH}_{H}}{\mathrm{THE}_{H}}$ > 0.1 |
| ***CHE 25 (<25% THE_H_)*** |  | 1. No CHE if $\frac{\mathrm{THEALTH}_{H}}{\mathrm{THE}_{H}}$ ≤ 0.25 2. CHE if $\frac{\mathrm{THEAlTH}_{H}}{\mathrm{THE}_{H}}$ > 0.25 |
| ***CHE 40 (<40% CTP_H_)*** |  | 1. No CHE if $\frac{\mathrm{THEALTH}_{H}}{\mathrm{CTP}_{H}}$ ≤ 0.4 2. CHE if $\frac{\mathrm{THEAlTH}_{H}}{\mathrm{CTP}_{H}}$ > 0.4 |
| **Key independent variables** | | |
| ***Number of physical conditions*** | HT002-HT008 HT010: Has any health professional ever told you that you have…?   - Hypertension or high blood pressure - Diabetes or high blood sugar - Cancer or a malignant tumor - Chronic lung disease such as asthma, chronic obstructive pulmonary disease/Chronic bronchitis or other chronic lung problems - Chronic heart diseases such as Coronary heart disease (heart attack or Myocardial Infarction), congestive heart failure, or other chronic heart problems - Stroke - Arthritis or rheumatism, Osteoporosis or other bone/joint diseases - High cholesterol   HT009: Has any health professional ever told you that you have any neurological, or psychiatric problems such as depression , Alzheimer’s/Dementia, unipolar/bipolar disorders, convulsions, Parkinson’s etc.?  HT009a [Ask only if HT009=1]: Which type of neurological or psychiatric problem(s) have you been diagnosed with?   1. Depression 2. Alzheimer’s disease, Dementia 3. Psychiatric problems such as unipolar/bipolar disorder, schizophrenia etc. 4. Neurological problems such as neuropathy, convulsions, migraine, Parkinson’s etc. 5. Other, please specify _____   HT011: Have you ever been diagnosed with any of the following chronic conditions or diseases?   1. Thyroid disorder   HT012. Have you ever been diagnosed with any of the following urogenital conditions or diseases?   - Chronic Renal Failure - Kidney Stones - BPH (Benign Prostatic Hyperplasia) | Counting variable (0-11; regrouped to 0,1,2,3+), including:   - Conditions under HT002-Ht008, HT010 - Neurological diseases (i.e. Alzheimer´s or other such as Parkinson`s): HT009=Yes and HT009a=b or d - Thyroid disorder: HT011a - Chronic kidney diseases (HT012: Chronic renal failure, kidney stones, BPH) |
| CESD-10 | FS701-FS710: Now think about the past week and the feelings you have experienced. Please tell me if each of the following was true for you much of the time during the past week.   - How often did you have trouble concentrating? - How often did you feel depressed? - How often did you feel tired or low in energy? - How often were you afraid of something? - How often did you feel you were overall satisfied? - How often did you feel alone? - How often were you bothered by things that don’t usually bother you? - How often did you feel that everything you did was an effort? - How often did you feel hopeful about the future? - How often did you feel happy? | Answer options:  1 Rarely or never (less than 1 day)  2 Sometimes (1 or 2 days)  3 Often (3 or 4 days)  4 Most or all of the time (5-7 days)  Score generated from 0 to 10:   - FS701-FS704 FS706-FS708: 1 2= 0; 3 4 =1 - FS705 FS709 FS710: 1 2=1; 3 4=0  1. No depressive symptoms (score < 4) 2. Depressive symptoms (score ≥ 4) |
| Screening question CIDI-SF dysphoria symptoms | MH201: During the last 12 months, was there ever a time when you felt sad, blue, or depressed for two weeks or more in a row?  MH202: [Ask only If MH201=1] Please think of the two-week period during the last 12 months when these feelings were worst. During that time did the feelings of being sad, blue, or depressed usually last all day long, most of the day, about half the day, or less than half the day?  MH203: [Ask If MH202=1 or 2] During those two weeks, did you feel this way every day, almost every day, or less often than that? | MH201:  1 Yes  2 No  MH202:  1 All day long  2 Most of the day  3 About half the day  4 Less than half the day  MH203:  1 Every day  2 Almost every day  3 Less often   1. Fail screening question (MH201 = 0 or MH202 = 3 4 or MH203 = 1 2) 2. Pass screening question (MH201 = 1 & MH202 = 1 2 & MH203 = 1 2) |
| Symptom questions CIDI-SF dysphoria | MH204-MH211: [Ask If MH203=1 or 2] Thinking about those same two weeks,   - Did you lose interest in most things? - Did you ever feel more tired out or low in energy than is usual for you? - Did you lose your appetite? - During the same two-week period did you have a lot more trouble concentrating than usual? - People sometimes feel down on themselves, and no good or worthless. During that two-week period, did you feel this way? - Did you think a lot about death – either your own, someone else’s, or death in general – during those two weeks? - Did you have more trouble falling asleep than you usually do during those two weeks? | Answer options:  Yes  No  Score generated from 0-7: Yes=1, No=0   1. No depressive symptoms dysphoria (screening questions failed or screening questions passed & score < 3) 2. Depressive symptoms dysphoria (Screening questions passed & score ≥ 3) |
| Screening questions CIDI-SF anhedonia | MH214: During the past 12 months, was there ever a time lasting two weeks or more when you lost interest in most things like hobbies, work, or activities that usually give you pleasure?  MH215: [Ask only if MH214=1] Please think of the two-week period during the last 12 months when you had the most complete loss of interest in things. During that two-week period, did the loss of interest usually last all day long, most of the day, about half the day, or less than half the day?  MH216: [Ask if MH215 = 1 or 2] Did you feel this way every day, almost every day, or less often during the two weeks? | MH214:  1 Yes  2 No  MH215:  1 All day long  2 Most of the day  3 About half the day  4 Less than half the day  MH216:  1 Every day  2 Almost every day  3 Less often   1. Fail screening question (MH214 = 0 or MH215 = 3 4 or MH216 = 1 2) 2. Pass screening question (MH214 = 1 & MH215 = 1 2 & MH216 = 1 2) |
| Symptom questions CIDI-SF anhedonia | MH217-MH222 [Ask if MH216 = 1 or 2] During those two weeks, did you experience the following?   - Did you feel tired out or low on energy all the time? - During those same two weeks, did you lose your appetite? - During those two weeks, did you have more trouble concentrating than usual? - People sometimes feel down on themselves, no good or worthless. Did you feel this way during that two-week period? - Did you think a lot about death during those two weeks – either your own, someone else’s, or death in general? - During those same two weeks, did you have more trouble falling asleep than you usually do? | Answer options:  Yes  No  Score generated from 0-7: Yes=1, No=0; one point when passing screening questions)   1. No depressive symptoms anhedonia (screening questions failed or screening questions passed & score < 3) 2. Depressive symptoms anhedonia (screening questions passed & score ≥ 3) |
| Self-reported depression or other psychiatric disorder | HT009: Has any health professional ever told you that you have any neurological, or psychiatric problems such as depression, Alzheimer’s/Dementia, unipolar/bipolar disorders, convulsions, Parkinson’s etc.?  HT009a [Ask only if HT009=1]: Which type of neurological or psychiatric problem(s) have you been diagnosed with?   1. Depression 2. Alzheimer’s disease, Dementia 3. Psychiatric problems such as unipolar/bipolar disorder, schizophrenia etc. 4. Neurological problems such as neuropathy, convulsions, migraine, Parkinson’s etc. 5. Other, please specify _____ | 1. No (HT009=No or HT009=Yes & HT009a≠a or c) 2. Yes (HT009=Yes & HT009a=a or c) |
| ***Mental health condition*** |  | 1. No mental health condition (CESD-10, CIDI-SF dysphoria and anhedonia & self-reported mental health condition = No) 2. Mental health condition (any CESD-10, CIDI-SF dysphoria and anhedonia & self-reported mental health condition = Yes) |
| ***Ownership of health insurance*** | HC102: Are you covered by health insurance? | 1. No health insurance 2. Owned health insurance |
| **Covariates** | | |
| ***Sex*** | DM003: [Instruction for the interviewer: Record sex of the respondent. If not clear to the interviewer, please ask the respondent about his/her sex.] | 1. Male 2. Female |
| ***Age*** | DM005: How old were you at your last birthday? | 1. 45-59 years 2. 60-74 years 3. 75+ years |
| ***Residence*** | Residence: provided in cover screen | 1. Rural 2. Urban |
| ***Marital status*** | DM021: What is your current (latest) marital status? | 1. Not married or in a relationship (Widowed, Divorced, Separated, Deserted, Never married) 2. Married or in a relationship (Currently married, Live-in relationship) |
| ***Religion*** | DM010: What is your religion? | 1. Hindu 2. Muslim 3. Christian 4. Sikh 5. Other or none (None, Buddhist/neo-Buddhist, Jain, Jewish, Parsi/Zoroastrian, Other) |
| ***Social group*** | DM012: What is your caste or tribe?  DM013: [Ask only if DM012< 3] Do you belong to a scheduled caste, a scheduled tribe, other backward class, or none of these? | DM012:  1 Caste, specifiy:  2 Tribe, specify:  3 No Caste/Tribe  4 Don’t know  DM013:  1 Scheduled caste  2 Scheduled tribe  3 Other backward class  4 None of them   1. Scheduled tribe (if DM012 <3 & DM013=2) 2. Scheduled caste (if DM012 <3 & DM013=1) 3. Other backward class (if DM012 <3 & DM013=3) 4. Other or no caste/tribe (if DM012 =3 or DM012 < 3 & DM013=4) |
| ***Educational level*** | DM006: Have you ever attended school?  DM008: [Ask only if DM006=1] What is the highest level of education that you completed? | DM006:  Yes  No  DM008:   1. Less than Primary school(Standard 1-4) 2. Primary school Completed (Standard 5-7) 3. Middle school Completed (Standard 8- 9) 4. Secondary School/Matriculation completed 5. Higher Secondary/Intermediate/Senior Secondary completed 6. Diploma and certificate holders 7. Graduate degree (B.A., B.Sc., B. Com.) completed 8. Post-graduate degree or (M.A., M.Sc., M. Com.) above (M.Phil, Ph.D.,Post-Doc) completed 9. Professional course/degree (B.Ed, BE, B.Tech, MBBS, BHMS, BAMS, B. Pharm, BCS, BCA, BBA, LLB, BVSc., B. Arch, M.Ed, ME, M.Tech, MD, M.Pharm, MCS, MCA, MBA, LLM, MVSc., M. Arch, MS, CA, CS, CWA) 10. No education (DM006=No) 11. Up to primary (DM008=1 2) 12. Middle school to higher secondary (DM008=3 4 5) 13. Diploma, graduation or above (DM008=6 7 8 9) |
| ***Employment status*** | WE001: Now, I am going to ask you some questions about your work and employment. Have you ever worked for at least 3 months during your lifetime? Work includes agricultural work, wage work, self-employed activities, and unpaid family business work. Work also includes all kinds of labor, excluding doing your own housework, whether you earn wages or not.  We004: Are you currently working? | 1. Never worked (if WE001=No) 2. Currently not working but worked before (if WE001=Yes & WE004=No) 3. Currently working (if WE001=Yes & WE004=Yes) |
| ***Household expenditure per capita*** | Total household expenditure (THE_H_)  CV001: Number of people living in household  Total household expenditure | - $\frac{{THE}_{H}}{CV001}$ - Quintiles generated   Q1 (the lowest) – Q5 (the highest) |

| **Table S2:** Missing values per variable | | |
| --- | --- | --- |
|  | Frequency | % |
| Physical condition | 230 | 0.4 |
| Mental condition | 144 | 0.2 |
| Outpatient care utilisation | 651 | 1.0 |
| Inpatient care utilisation | 679 | 1.0 |
| CHE 10% of THE_H_ | 2 | 0.0 |
| CHE 25% of THE_H_ | 2 | 0.0 |
| CHE 40% of CTP_H_ | 2 | 0.0 |
| Sex | 0 | 0.0 |
| Age | 0 | 0.0 |
| Residence | 0 | 0.0 |
| Marital status | 0 | 0.0 |
| Social group | 716 | 1.1 |
| Religion | 5 | 0.0 |
| Educational level | 1 | 0.0 |
| Employment status | 63 | 0.1 |
| Household expenditure per capita | 2 | 0.0 |
| Ownership of health insurance | 573 | 0.9 |
| CHE = catastrophic health expenditure; THEH = total household expenditure; CTPH = capacity to pay | | |

| **Table S3:** Number of variables with missing data per respondent | | |
| --- | --- | --- |
|  | Frequency | % |
| 0 | 63,161 | 97.6 |
| 1 | 1,176 | 1.8 |
| 2 | 18 | 0.0 |
| 3 | 364 | 0.6 |
| 4 | 8 | 0.0 |
| 5 | 136 | 0.2 |
| 6 | 9 | 0.0 |

| **Table S4:** Logistic regression models for healthcare utilisation | | | | | | | | | | | | | | | | | | | | | | | | | | | | | |  |  |  |
| --- | --- | --- | --- | --- | --- | --- | --- | --- | --- | --- | --- | --- | --- | --- | --- | --- | --- | --- | --- | --- | --- | --- | --- | --- | --- | --- | --- | --- | --- | --- | --- | --- |
|  | | Outpatient care utilisation | | | | | | | | | | |  | | | | Inpatient care utilisation | | | | | | | | | | | | |  |  |  |
|  | | OR [95% CI] | | | AOR [95% CI] | | | | | | | |  | | | | OR [95% CI] | | | | | | AOR [95% CI] | | | | | | |  |  |  |
| **Moderation *(*Mental health condition # Health insurance ownership # Number of physical conditions)** | | | | | | | | | | | | | | | |  | | | | | |  | | | | | | | |  |  |  |
| Physical conditions # No mental health condition # No health insurance | | 1.43 [1.35-1.52] | | | | 1.41 [1.33-1.49] | | | | | | | |  | | | 1.46 [1.37-1.56] | | | | | | 1.37 [1.28-1.47] | | | | | | |  |  |  |
| Physical conditions # No mental health condition # Owned health insurance | | 1.52 [1.40-1.66] | | | | 1.54 [1.42-1.66] | | | | | | | |  | | | 1.66 [1.52-1.81] | | | | | | 1.56 [1.43-1.70] | | | | | | |  |  |  |
| Physical conditions # With mental health condition # No health insurance | | 1.40 [1.33-1.48] | | | | 1.35 [1.28-1.42] | | | | | | | |  | | | 1.90 [1.70-2.13] | | | | | | 1.74 [1.60-1.89] | | | | | | |  |  |  |
| Physical conditions # With mental health condition # Owned health insurance | | 1.50 [1.38-1.63] | | | | 1.46 [1.35-1.59] | | | | | | | |  | | | 2.18 [1.89-2.52] | | | | | | 2.02 [1.76-2.31] | | | | | | |  |  |  |
| **Sex *(ref. Male)*** | |  | | | |  | | | | | | | |  | | |  | | | | | |  | | | | | | |  |  |  |
| Female | |  | | | | 1.42 [1.31-1.55] | | | | | | | |  | | |  | | | | | | 0.81 [0.66-0.99] | | | | | | |  |  |  |
| **Age *(ref. 45-59 years)*** | |  | | | |  | | | | | | | |  | | |  | | | | | |  | | | | | | |  |  |  |
| 60-74 years | |  | | | | 1.16 [1.08-1.25] | | | | | | | |  | | |  | | | | | | 0.95 [0.79-1.15] | | | | | | |  |  |  |
| 75+ years | |  | | | | 1.06 [0.93-1.20] | | | | | | | |  | | |  | | | | | | 0.92 [0.70-1.22] | | | | | | |  |  |  |
| **Place of residence *(ref. Rural)*** | |  | | | |  | | | | | | | |  | | |  | | | | | |  | | | | | | |  |  |  |
| Urban | |  | | | | 0.79 [0.73-0.86] | | | | | | | |  | | |  | | | | | | 0.96 [0.80-1.14] | | | | | | |  |  |  |
| **Marital status *(ref. Not married or in a relationship)*** | |  | | | |  | | | | | | | |  | | |  | | | | | |  | | | | | | |  |  |  |
| Married or in a relationship | |  | | | | 1.03 [0.95-1.11] | | | | | | | |  | | |  | | | | | | 1.08 [0.94-1.25] | | | | | | |  |  |  |
| **Religion *(ref. Hindu)*** | |  | | | |  | | | | | | | |  | | |  | | | | | |  | | | | | | |  |  |  |
| Muslim | |  | | | | 1.34 [1.18-1.53] | | | | | | | |  | | |  | | | | | | 1.26 [0.95-1.69] | | | | | | |  |  |  |
| Christian | |  | | | | 0.75 [0.65-0.85] | | | | | | | |  | | |  | | | | | | 1.01 [0.76-1.33] | | | | | | |  |  |  |
| Sikh | |  | | | | 1.72 [1.48-2.01] | | | | | | | |  | | |  | | | | | | 0.84 [0.63-1.13] | | | | | | |  |  |  |
| Other or none | |  | | | | 0.89 [0.72-1.09] | | | | | | | |  | | |  | | | | | | 1.27 [0.86-1.86] | | | | | | |  |  |  |
| **Social group *(ref. Scheduled tribe)*** | |  | | | |  | | | | | | | |  | | |  | | | | | |  | | | | | | |  |  |  |
| Scheduled caste | |  | | | | 1.66 [1.50-1.85] | | | | | | | |  | | |  | | | | | | 1.07 [0.85-1.36] | | | | | | |  |  |  |
| Other backward class | |  | | | | 1.44 [1.30-1.59] | | | | | | | |  | | |  | | | | | | 0.91 [0.73-1.14] | | | | | | |  |  |  |
| Other or no caste/tribe | |  | | | | 1.75 [1.56-1.97] | | | | | | | |  | | |  | | | | | | 0.91 [0.71-1.17] | | | | | | |  |  |  |
| **Educational level *(ref. No education)*** | |  | | | |  | | | | | | | |  | | |  | | | | | |  | | | | | | |  |  |  |
| Up to primary | |  | | | | 1.09 [1.01-1.18] | | | | | | | |  | | |  | | | | | | 0.99 [0.81-1.21] | | | | | | |  |  |  |
| Middle school to higher secondary | |  | | | | 0.95 [0.86-1.04] | | | | | | | |  | | |  | | | | | | 0.70 [0.55-0.88] | | | | | | |  |  |  |
| Diploma, graduation or above | |  | | | | 0.77 [0.61-0.97] | | | | | | | |  | | |  | | | | | | 0.33 [0.23-0.48] | | | | | | |  |  |  |
| **Employment status *(ref. Never worked)*** | |  | | | |  | | | | | | | |  | | |  | | | | | |  | | | | | | |  |  |  |
| Currently not working but worked before | |  | | | | 1.55 [1.41-1.71] | | | | | | | |  | | |  | | | | | | 1.72 [1.43-2.07] | | | | | | |  |  |  |
| Currently working | |  | | | | 1.39 [1.27-1.52] | | | | | | | |  | | |  | | | | | | 0.86 [0.72-1.04] | | | | | | |  |  |  |
| **Household expenditure per capita *(ref. Q1 (the lowest))*** | |  | | | |  | | | | | | | |  | | |  | | | | | |  | | | | | | |  |  |  |
| Q2 | |  | | | | 1.15 [1.06-1.25] | | | | | | | |  | | |  | | | | | | 1.14 [0.96-1.36] | | | | | | |  |  |  |
| Q3 | |  | | | | 1.30 [1.19-1.41] | | | | | | | |  | | |  | | | | | | 1.54 [1.29-1.84] | | | | | | |  |  |  |
| Q4 | |  | | | | 1.37 [1.25-1.50] | | | | | | | |  | | |  | | | | | | 1.89 [1.56-2.29] | | | | | | |  |  |  |
| Q5 (the highest) | |  | | | | 1.26 [1.12-1.42] | | | | | | | |  | | |  | | | | | | 2.58 [2.04-3.28] | | | | | | |  |  |  |
| Note: Estimates are weighted \| ref. = reference group | |  | | | |  | | | | | | | |  | | |  | | | | | |  | | | | | | |  |  |  |
|  | |  | | | |  | | | | | | | |  | | |  | | | | | |  | | | | | | |  |  |  |
| **Table S5:** Logistic regression models for the experience of catastrophic health expenditure | | | | | | | | | | | | | | | | | | | | | | | | | | | | | | | | |
|  | CHE (>10% of THE_H_) | | | | | |  | | | CHE (>25% of THE_H_) | | | | | | | | | | | | | |  | | | CHE (>40% of CTP_H_) | | | | | |
|  | OR [95% CI] | | AOR [95% CI] | | | | |  | | | OR [95% CI] | | | | | | | AOR [95% CI] | | | | | | |  | | OR [95% CI] | | | | AOR [95% CI] | |
| **Moderation *(*Mental health condition # Health insurance ownership # Number of physical conditions)** | | | | | | | | | | | | | | |  | | | | |  |  | | | | | | | |  | | | |
| Physical conditions # No mental health condition # No health insurance | 1.25 [1.18-1.33] | | | 1.20 [1.14-1.27] | | | | |  | | | 1.21 [1.14-1.29] | | | | | | | 1.11 [1.05-1.18] | | | | | | |  | | 1.13 [1.07-1.19] | | | | 1.10 [1.05-1.16] |
| Physical conditions # No mental health condition # Owned health insurance | 1.18 [1.06-1.31] | | | 1.14 [1.04-1.25] | | | | |  | | | 1.13 [1.04-1.23] | | | | | | | 1.04 [0.96-1.13] | | | | | | |  | | 1.02 [0.95-1.10] | | | | 1.02 [0.95-1.10] |
| Physical conditions # With mental health condition # No health insurance | 1.53 [1.45-1.62] | | | 1.46 [1.38-1.53] | | | | |  | | | 1.54 [1.46-1.63] | | | | | | | 1.41 [1.33-1.49] | | | | | | |  | | 1.48 [1.40-1.56] | | | | 1.40 [1.33-1.48] |
| Physical conditions # With mental health condition # Owned health insurance | 1.37 [1.25-1.49] | | | 1.34 [1.23-1.45] | | | | |  | | | 1.35 [1.23-1.49] | | | | | | | 1.27 [1.15-1.39] | | | | | | |  | | 1.31 [1.20-1.44] | | | | 1.29 [1.18-1.41] |
| **Sex *(ref. Male)*** |  | | |  | | | | |  | | |  | | | | | | |  | | | | | | |  | |  | | | |  |
| Female |  | | | 1.08 [0.98-1.20] | | | | |  | | |  | | | | | | | 1.09 [0.97-1.23] | | | | | | |  | |  | | | | 1.06 [0.96-1.17] |
| **Age *(ref. 45-59 years)*** |  | | |  | | | | |  | | |  | | | | | | |  | | | | | | |  | |  | | | |  |
| 60-74 years |  | | | 1.19 [1.10-1.28] | | | | |  | | |  | | | | | | | 1.35 [1.21-1.50] | | | | | | |  | |  | | | | 1.45 [1.33-1.59] |
| 75+ years |  | | | 1.47 [1.29-1.67] | | | | |  | | |  | | | | | | | 1.51 [1.26-1.81] | | | | | | |  | |  | | | | 1.65 [1.43-1.92] |
| **Place of residence *(ref. Rural)*** |  | | |  | | | | |  | | |  | | | | | | |  | | | | | | |  | |  | | | |  |
| Urban |  | | | 0.67 [0.61-0.73] | | | | |  | | |  | | | | | | | 0.62 [0.55-0.69] | | | | | | |  | |  | | | | 0.59 [0.53-0.64] |
| **Marital status *(ref. Not married or in a relationship)*** |  | | |  | | | | |  | | |  | | | | | | |  | | | | | | |  | |  | | | |  |
| Married or in a relationship |  | | | 1.40 [1.29-1.52] | | | | |  | | |  | | | | | | | 1.38 [1.22-1.56] | | | | | | |  | |  | | | | 1.33 [1.20-1.47] |
| **Religion *(ref. Hindu)*** |  | | |  | | | | |  | | |  | | | | | | |  | | | | | | |  | |  | | | |  |
| Muslim |  | | | 1.38 [1.17-1.62] | | | | |  | | |  | | | | | | | 1.01 [0.87-1.17] | | | | | | |  | |  | | | | 1.23 [1.09-1.38] |
| Christian |  | | | 0.79 [0.69-0.91] | | | | |  | | |  | | | | | | | 0.83 [0.68-1.02] | | | | | | |  | |  | | | | 0.80 [0.67-0.96] |
| Sikh |  | | | 1.00 [0.86-1.16] | | | | |  | | |  | | | | | | | 0.80 [0.65-0.98] | | | | | | |  | |  | | | | 0.85 [0.72-1.02] |
| Other or none |  | | | 1.04 [0.84-1.28] | | | | |  | | |  | | | | | | | 1.21 [0.90-1.64] | | | | | | |  | |  | | | | 1.19 [0.93-1.52] |
| **Social group *(ref. Scheduled tribe)*** |  | | |  | | | | |  | | |  | | | | | | |  | | | | | | |  | |  | | | |  |
| Scheduled caste |  | | | 1.85 [1.65-2.08] | | | | |  | | |  | | | | | | | 1.62 [1.37-1.93] | | | | | | |  | |  | | | | 1.58 [1.38-1.82] |
| Other backward class |  | | | 1.54 [1.38-1.72] | | | | |  | | |  | | | | | | | 1.39 [1.18-1.63] | | | | | | |  | |  | | | | 1.27 [1.11-1.45] |
| Other or no caste/tribe |  | | | 1.76 [1.55-1.99] | | | | |  | | |  | | | | | | | 1.54 [1.29-1.83] | | | | | | |  | |  | | | | 1.45 [1.25-1.67] |
| **Educational level *(ref. No education)*** |  | | |  | | | | |  | | |  | | | | | | |  | | | | | | |  | |  | | | |  |
| Up to primary |  | | | 0.97 [0.89-1.06] | | | | |  | | |  | | | | | | | 1.03 [0.92-1.17] | | | | | | |  | |  | | | | 0.89 [0.81-0.99] |
| Middle school to higher secondary |  | | | 0.93 [0.83-1.03] | | | | |  | | |  | | | | | | | 0.86 [0.75-0.98] | | | | | | |  | |  | | | | 0.79 [0.71-0.89] |
| Diploma, graduation or above |  | | | 0.73 [0.54-0.98] | | | | |  | | |  | | | | | | | 0.54 [0.42-0.68] | | | | | | |  | |  | | | | 0.52 [0.42-0.64] |
| **Employment status *(ref. Never worked)*** |  | | |  | | | | |  | | |  | | | | | | |  | | | | | | |  | |  | | | |  |
| Currently not working but worked before |  | | | 1.24 [1.13-1.37] | | | | |  | | |  | | | | | | | 1.20 [1.06-1.37] | | | | | | |  | |  | | | | 1.12 [1.00-1.25] |
| Currently working |  | | | 1.05 [0.94-1.17] | | | | |  | | |  | | | | | | | 0.91 [0.79-1.04] | | | | | | |  | |  | | | | 0.93 [0.83-1.05] |
| **Household expenditure per capita *(ref. Q1 (the lowest))*** |  | | |  | | | | |  | | |  | | | | | | |  | | | | | | |  | |  | | | |  |
| Q2 |  | | | 1.40 [1.28-1.53] | | | | |  | | |  | | | | | | | 1.40 [1.19-1.64] | | | | | | |  | |  | | | | 1.21 [1.08-1.35] |
| Q3 |  | | | 1.89 [1.72-2.06] | | | | |  | | |  | | | | | | | 2.17 [1.87-2.52] | | | | | | |  | |  | | | | 1.64 [1.47-1.83] |
| Q4 |  | | | 1.97 [1.79-2.16] | | | | |  | | |  | | | | | | | 2.88 [2.47-3.35] | | | | | | |  | |  | | | | 1.58 [1.40-1.77] |
| Q5 (the highest) |  | | | 2.42 [2.14-2.74] | | | | |  | | |  | | | | | | | 4.42 [3.79-5.17] | | | | | | |  | |  | | | | 1.99 [1.75-2.26] |
| Note: Estimates are weighted. \| CHE = catastrophic health expenditure; THE_H_ = total household expenditure; CTP_H_ = capacity to pay; ref. = reference group | | | | | | | | | | | | | | | | | | | | | | | | | | | | | | | | |

| **Table S6:** Results of the Wald test between stratification groups for the estimates of the logistic regressions | | | | | | | | | | |
| --- | --- | --- | --- | --- | --- | --- | --- | --- | --- | --- |
| Physical conditions # | **Healthcare utilisation** | | | | **Experience of CHE** | | | | | |
|  | **Outpatient** | | **Inpatient** | | **>10% of THE_H_** | | **>25% of THE_H_** | | **>40 % of CTP_H_** | |
|  | Chi2 | p-value | Chi2 | p-value | Chi2 | p-value | Chi2 | p-value | Chi2 | p-value |
| No mental health condition#No health insurance vs. No mental health condition#Owned health insurance | 3.46 | 0.0628 | 7.92 | 0.0049 | 0.91 | 0.3410 | 2.18 | 0.1396 | 3.90 | 0.0483 |
| No mental health condition#No health insurance vs. With mental health condition#No health insurance | 1.63 | 0.2011 | 21.48 | <0.0001 | 33.18 | <0.0001 | 41.96 | <0.0001 | 57.27 | <0.0001 |
| No mental health condition#No health insurance vs. With mental health condition#Owned health insurance | 0.61 | 0.4361 | 31.13 | <0.0001 | 5.04 | 0.0248 | 6.00 | 0.0143 | 10.30 | 0.0013 |
| No mental health condition#Owned health insurance vs. With mental health condition#No health insurance | 8.12 | 0.0044 | 3.40 | 0.0651 | 20.79 | <0.0001 | 43.15 | <0.0001 | 61.40 | <0.0001 |
| No mental health condition#Owned health insurance vs. With mental health condition#Owned health insurance | 0.81 | 0.3668 | 12.04 | 0.0005 | 6.57 | 0.0104 | 10.98 | 0.0009 | 18.45 | <0.0001 |
| With mental health condition#No health insurance vs. With mental health condition#Owned health insurance | 2.97 | 0.0849 | 3.83 | 0.0504 | 3.22 | 0.0727 | 4.30 | 0.0382 | 3.11 | 0.0776 |
| CHE = catastrophic health expenditure; THE_H_ = total household expenditure; CTP_H_ = capacity to pay | | | | | | | | | | |

| **Table S7:** Predicted probabilities for healthcare utilisation and experience of catastrophic health expenditure | | | | | | | |
| --- | --- | --- | --- | --- | --- | --- | --- |
| Ownership of health insurance | Mental health condition | Number of physical conditions | Outpatients  probability  [95% CI] | Inpatients  Probability  [95% CI] | CHE (>10% of THE_H_)  [95% CI] | CHE (>25% of THE_H_) Probability  [95% CI] | CHE (>40 % of CTP_H_) Probability  [95% CI] |
| No health insurance | No mental health condition | 0 | 48.9  [48.0-49.9] | 3.9  [3.6-4.2] | 27.5  [26.6-28.3] | 9.6  [9.1-10.1] | 15.4  [14.7-16.1] |
|  |  | 1 | 58.1  [57.0-59.3] | 5.7  [5.3-6.0] | 32.1  [31.1-33.1] | 11.3  [10.8-11.9] | 17.2  [16.6-17.8] |
|  |  | 2 | 66.4  [64.2-68.6] | 8.3  [7.5-9.0] | 37.4  [35.3-39.6] | 13.7  [12.6-14.8] | 19.0  [17.7-20.2] |
|  |  | 3+ | 73.3  [70.3-76.4] | 11.2  [9.7-12.8] | 41.8  [38.2-45.4] | 15.8  [13.8-17.7] | 20.4  [18.3-22.4] |
|  | With mental health condition | 0 | 49.9  [49.0-50.9] | 4.1  [3.8-4.5] | 27.8  [26.9-28.6] | 9.9  [9.3-10.4] | 16.0  [15.3-16.7] |
|  |  | 1 | 58.1  [56.8-59.3] | 7.3  [6.6-8.0] | 36.5  [35.3-37.6] | 14.0  [13.3-14.7] | 21.6  [20.7-22.5] |
|  |  | 2 | 65.2  [62.9-67.4] | 13.6  [11.1-16.1] | 47.0  [44.7-49.3] | 20.3  [18.7-22] | 28.4  [26.5-30.3] |
|  |  | 3+ | 72.2  [69.3-75.2] | 21.3  [17.4-25.2] | 57.2  [53.9-60.6] | 27.6  [24.7-30.5] | 36.9  [33.8-40.0] |
| With health insurance | No mental health condition | 0 | 47.5  [46.5-48.5] | 3.9  [3.5-4.2] | 26.2  [25.4-27.0] | 9.2  [8.7-9.7] | 14.4  [13.8-15.0] |
|  |  | 1 | 58.9  [57.1-60.7] | 6.4  [5.9-6.9] | 30.2  [28.2-32.1] | 10.6  [9.9-11.3] | 15.5  [14.7-16.4] |
|  |  | 2 | 68.9  [65.5-72.4] | 10.1  [8.8-11.4] | 34.4  [29.5-39.4] | 11.7  [10.3-13.2] | 15.6  [14.0-17.2] |
|  |  | 3+ | 78.3  [74.5-82.1] | 16.3  [13.4-19.2] | 39.1  [32.4-45.7] | 14.0  [11.5-16.5] | 17.3  [14.7-20.0] |
|  | With mental health condition | 0 | 48.8  [47.8-49.8] | 4.1  [3.7-4.4] | 27.1  [26.3-27.9] | 9.6  [9.1-10.1] | 15.3  [14.6-16.0] |
|  |  | 1 | 58.7  [56.8-60.6] | 8.2  [7.2-9.2] | 33.3  [31.5-35.1] | 12.4  [11.4-13.4] | 19.4  [18.1-20.8] |
|  |  | 2 | 68.9  [65.5-72.2] | 16.7  [13.3-20.1] | 42.5  [38.6-46.3] | 17.2  [14.8-19.6] | 24.8  [21.8-27.9] |
|  |  | 3+ | 76.4  [72.1-80.6] | 30.0  [22.2-37.8] | 48.5  [42.5-54.5] | 20.1  [16.0-24.2] | 28.4  [23.4-33.4] |
| Note: Estimates are weighted and adjusted for: age, sex, place of residence, marital status, religion, social group, education, employment status, household expenditure per capita \| CHE = catastrophic health expenditure THE_H_ = total household expenditure; CTP_H_ = capacity to pay | | | | | | | |
